# Nitrogen use efficiency of flue−cured tobacco genotypes: physiological basis and relative contributions of nitrogen uptake and utilization

**DOI:** 10.3389/fpls.2026.1727901

**Published:** 2026-03-11

**Authors:** Weiguo Ye, Jia Lei, Xianyun Zhong, Changyue Qi, Najam-Ud- Din, Yuanyuan Wang, Huaiyuan Li, Jianjun Chen, Shiyuan Deng

**Affiliations:** 1 College of Agriculture, South China Agricultural University, Guangzhou, China; 2 China Tobacco Guangdong Industrial Company Limited, Guangzhou, China; 3 Basic Experimental and Practical Training Center, South China Agricultural University, Guangzhou, China

**Keywords:** flue−cured tobacco, genotype, nitrogen uptake efficiency (NUpE), nitrogen use efficiency (NUE), nitrogen utilization efficiency (NUtE)

## Abstract

Understanding the physiological processes that regulate nitrogen uptake efficiency (NUpE), nitrogen utilization efficiency (NUtE), and nitrogen use efficiency (NUE) in crops is essential for developing nitrogen-efficient varieties. We conducted a two−year field study (2021–2022, Shixing, southern China) comparing three flue−cured tobacco genotypes (Yunyan 87, Yueyan 97, K326) under two nitrogen application rates: traditional (150 kg ha^-1^) and reduced (105 kg ha^-1^, −30%). Across both rates, the high−NUpE genotypes (Yueyan 97, K326) showed substantially greater root biomass, length, surface area, volume, vigor, bleeding sap and nitrate flow rates, and higher activities of key N−metabolism enzymes than low−NUpE Yunyan 87. These root and physiological traits were positively correlated with nitrogen uptake efficiency, indicating that they are major correlates of NUpE and may contribute to its variation. Compared with Yunyan 87 and K326 (both low−NUtE genotypes), Yueyan 97 (a high−NUtE genotype) exhibited a significantly lower respiratory rate despite a lower net photosynthetic rate. This pattern is consistent with higher NUtE being associated with a balance between net photosynthesis and respiration that favors reduced respiratory consumption. Path analysis indicated strong conditional associations of both NUpE and NUtE with NUE across genotypes. While path coefficients do not imply causality, the results suggest that jointly improving NUpE and NUtE may be a promising avenue for achieving high yield and improved NUE in flue-cured tobacco. In conclusion, this study identifies physiological traits that are strongly associated with NUpE and NUtE in flue-cured tobacco and provides insights to guide future efforts aimed at enhancing NUE in this crop.

## 1 Introduction

Nitrogen (N) is an essential nutrient for plant growth and plays a vital role in plant development. However, nitrogen use efficiency (NUE) in crop plants remains low. For major crops, the average NUE is only about 30%–35% (Iqbal et al., 2019). Field studies, including ^15^N tracer experiments, shows that flue-cured tobacco similarly exhibits low fertilizer N use efficiency. Seasonal and whole-plant uptake efficiencies are often roughly 25–30% and may decline further following heavy rainfall (Wang et al., 2018; Ding et al., 2024; Zhang et al., 2025). A substantial fraction of applied fertilizer N may be lost via leaching, ammonia volatilization, or denitrification, contributing to water eutrophication and air pollution (Chang et al., 2021; McDowell et al., 2021). Therefore, enhancing NUE in crops and reducing nitrogen fertilizer inputs in agricultural practices have become critical objectives for achieving a sustainable balance between crop yield, farm profitability, and environmental protection.

According to Moll et al. (1982), nitrogen use efficiency (NUE) is crop yield per unit of available N (soil and/or fertilizer). NUE = NUpE × NUtE, where NUpE is the fraction of supplied N taken up by the plant and NUtE is the yield produced per unit N taken up. Extensive studies show that the relative contributions of NUpE and NUtE to overall NUE depend on crop genotype, N application rate and environmental conditions (Stahl et al., 2019; Huang et al., 2025; Zi et al., 2025). Thus, breeding and agronomic management should target both components to achieve stable yields with reduced N inputs.

NUpE is generally associated with crop root biomass, morphology, and activity (Vazquez-Carrasquer et al., 2021; Ranjan et al., 2023). Total absorptive surface area, active absorptive area, and root vigor affect a crop’s ability to acquire soil nitrogen (Zhang et al., 2020; Anandan et al., 2022). Root depth and spatial distribution influence crop accessibility to mineral nitrogen across different soil layers (Lynch, 2019). Root uptake kinetics and the expression of inorganic nitrogen transporters further regulate uptake efficiency (Sandhu et al., 2021; Huang et al., 2023; Raimanová et al., 2024). In addition, irrigation, rainfall, and soil moisture can alter nitrogen leaching and root traits, thereby affecting NUpE (Porte et al., 2023; Li et al., 2025a, b). Therefore, evaluating these traits under realistic field conditions is essential.

Research on NUtE highlights the coordinated allocation of carbon and nitrogen metabolism within plants. Photosynthetic nitrogen use efficiency (PNUE) fundamentally reflects the carbon fixation capacity per unit leaf nitrogen (Zhong et al., 2019). Consequently, PNUE, associated leaf photosynthetic parameters, and the strength of leaf carbon–nitrogen coupling are typically positively correlated with NUtE (Ji et al., 2020; Flores-Saavedra et al., 2024; Mahboob et al., 2024). At the same time, optimizing nitrogen allocation in the photosynthetic apparatus increases the photosynthetic rate and thereby improves NUtE (Jian et al., 2024). Additionally, respiratory costs diminish net photosynthetic carbon gain, thereby lowering NUtE (Amthor, 2025). Delaying senescence can extend the duration of leaf photosynthesis. In parallel, internal nitrogen remobilization reallocates nitrogen from senescing leaves to grains or newly formed leaves, supporting yield formation and ultimately shaping NUtE (Mirosavljević et al., 2020; Guo et al., 2021). However, in leaf crops (e.g., tobacco), delayed senescence can alter leaf chemical composition and market quality, resulting in trade-offs between yield and quality (Zhou et al., 2018).

Despite growing interest in flue-cured tobacco, most studies still emphasize genotypic variation in NUE or whole-plant nitrogen uptake, with limited differentiation of the processes associated with NUpE and NUtE (Zhong et al., 2017; Fan et al., 2018; Andrade et al., 2020; Feng et al., 2024b). Moreover, relative to cereals, mechanistic research on NUE in tobacco remains limited for several reasons. First, tobacco is harvested for leaves rather than grain. As a result, grain-based definitions of NUpE and NUtE do not translate well, undermining methodological consistency and cross-study comparability. Second, empirical evidence linking N uptake, xylem loading and transport, assimilation and reassimilation, photosynthesis, respiratory costs, and leaf biomass formation is sparse. Third, key NUtE-related processes in tobacco (photosynthesis, respiration, N–C coupling) are less quantitatively characterized than in cereals. Finally, because many studies use controlled systems, field validation of efficiency gains remains limited. This further constrains actionable breeding and management targets.

**Figure 1:**
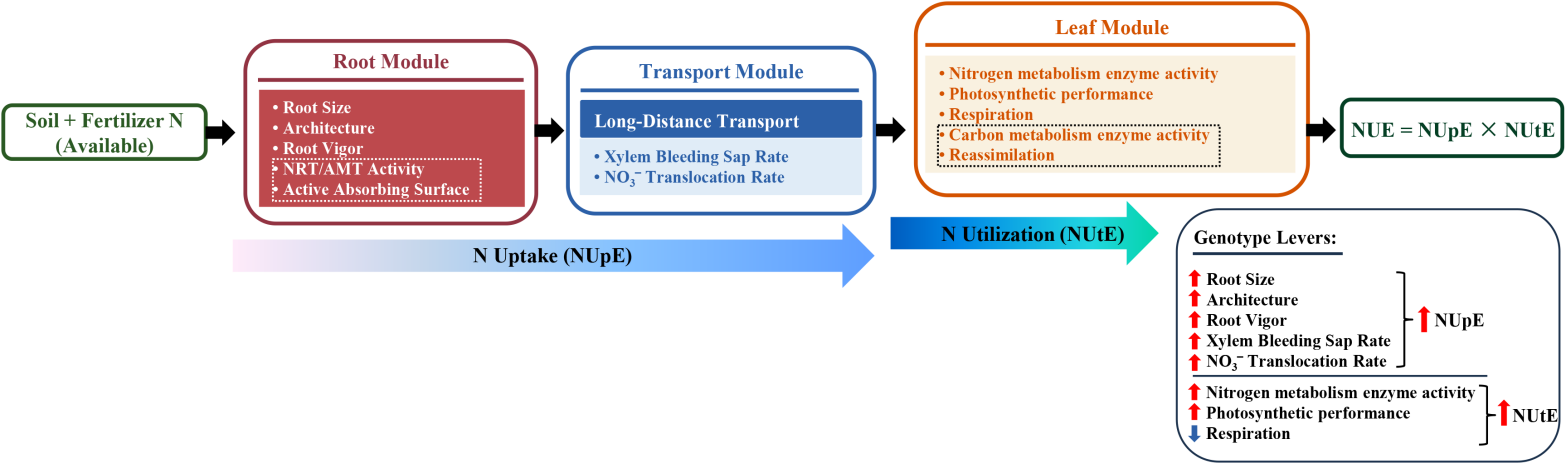
Conceptual framework and hypotheses for flue-cured tobacco NUE. All indicators are presented in this article except those within the dashed boxes. N, nitrogen; NRT, nitrate transporter; AMT, ammonium transporter; NUpE, nitrogen uptake efficiency; NUtE, nitrogen utilization efficiency; NUE, nitrogen use efficiency.

Based on the above insights and the identified research gaps, we propose a trait module framework for flue-cured tobacco to elucidate key components of NUE: (i) root phenotypes (overall size, architecture, and physiological activity) are closely associated with NUpE; (ii) xylem loading and long-distance transport are closely associated with the supply of inorganic N to leaves; (iii) leaf assimilation capacity, photosynthetic potential, and respiratory metabolic costs are closely associated with variation in NUtE (**Figure 1**). To test these hypotheses under field conditions, we conducted field experiments in the flue-cured tobacco production area of Shaoguan. We evaluated three commonly used genotypes (Yunyan 87, Yueyan 97, and K326) under two nitrogen application rates (the local traditional rate and a 30% reduced rate). We compared NUE, NUpE, and NUtE among the three genotypes and measured relevant root and leaf physiological traits. We then analyzed the relationships between these traits and nitrogen efficiency metrics. Our goal was to identify the physiological processes linked to NUpE and NUtE in flue-cured tobacco. We also aimed to provide mechanistic, field-verifiable evidence to guide the selection of genotypes with high nitrogen efficiency.

## 2 Materials and methods

### 2.1 Experimental station

In 2021 and 2022, field experiments were conducted in Anshui Village, Mashi Town, Shixing County, Shaoguan City, Guangdong Province, China (25°03′ N, 114°16′ E). This area has a subtropical monsoon climate. The predominant cropping system was a rotation between flue−cured tobacco and rice, allowing two harvests per year. Typically, flue−cured tobacco was transplanted in late February and harvested from late May to early July. The soil in the experimental field were classified as clay loam (Schad, 2023). Soil samples (0–20 cm) were collected from five randomly selected sampling points within the field, air−dried, and sieved through an 80−mesh sieve. The sieved samples were analyzed for soil pH (Nicol et al., 2008), organic matter (Tian et al., 2025), total nitrogen (Bremner and Tabatabai, 2008), total phosphorus (Taylor, 2008), total potassium (Han et al., 2024), alkali−hydrolyzable nitrogen (Greenfield, 2001), available phosphorus (Wu et al., 2024), and available potassium (Rupngam et al., 2025). For the 0–20 cm soil layer, the basic physicochemical properties in 2021 were as follows: pH 5.40; organic matter 21.31 g kg^−1^; total nitrogen 1.30 g kg^−1^; total phosphorus 0.77 g kg^−1^; total potassium 10.23 g kg^−1^; alkali−hydrolyzable nitrogen 121.60 mg kg^−1^; available phosphorus 65.76 mg kg^−1^; and available potassium 187.54 mg kg^−1^. The corresponding values in 2022 were: pH 5.65; organic matter 16.78 g kg^−1^; total nitrogen 1.20 g kg^−1^; total phosphorus 0.60 g kg^−1^; total potassium 12.02 g kg^−1^; alkali−hydrolyzable nitrogen 108.69 mg kg^−1^; available phosphorus 48.99 mg kg^−1^; and available potassium 215.60 mg kg^−1^.

### 2.2 Experimental design

In this study, flue−cured tobacco (*Nicotiana tabacum* L.) included three local genotypes: Yunyan 87, Yueyan 97 and K326. These genotypes were grown under two N application rates: a traditional rate (150 kg N ha^−1^) and a 30% reduced rate (105 kg N ha^−1^). A control (0 kg N ha^−1^) was also included for each genotype to assess fertilizer nitrogen use efficiency. Field experiments were conducted in 2021 and 2022, using a randomized complete block design with three replicates. Each experimental plot covered approximately 40.32 m² and comprised four rows with 15 plants per row (60 plants per plot). Row spacing was 1.20 m and intra−row spacing was 0.56 m. In addition to N, phosphorus (P_2_O_5_) was applied at 60 kg ha^−1^ and potassium (K_2_O) at 473 kg ha^−1^. All P was applied as basal fertilizer, whereas N and K were split between basal and topdressing in a 60:40 ratio. Throughout the growing season, weeds, pests and diseases were effectively controlled to ensure optimal growth conditions for the flue−cured tobacco.

### 2.3 Experimental sampling and measurements

#### 2.3.1 Yield and nitrogen−use characteristics

In each experimental plot, 20 uniformly growing plants were randomly selected. When they reached the defined maturity standards, these plants were harvested and cured in batches according to standard local practice. The cured−leaf yield was subsequently recorded. During the maturity stage, three additional plants were randomly sampled from each plot and separated into root, stem and leaf components. The samples were subjected to a kill−green treatment in an oven at 105 °C for 30 min, and then oven−dried at 80 °C to constant weight. After obtaining the dry weight, the samples were ground and sieved through an 80−mesh sieve. Total nitrogen concentration was determined by the Kjeldahl method using a Kjeltec™ 8400 analyzer (Foss Analytical A/S, Hillerød, Denmark) (Bremner and Tabatabai, 2008). Nitrogen accumulation in each organ was calculated as organ dry weight × organ N concentration. Total plant N accumulation was then obtained as the sum of root, stem and leaf N accumulation. Soil N supply was estimated following the methods described in Soil and Fertilizer Science (2nd ed.) (Lu and Xie, 2011). Total N supply was defined as the sum of soil N supply and applied fertilizer N. Nitrogen use efficiency (NUE), nitrogen uptake efficiency (NUpE), nitrogen utilization efficiency (NUtE), and agronomic nitrogen use efficiency (aNUE) were calculated according to Moll et al. (1982) and Novoa and Loomis (1981) as follows:


$$NUE\;=\;\frac{Leaf\;dry\;weight}{Total\;nitrogen\;supply}$$

$$NUpE\;=\;\frac{Plant\;nitrogen\;accumulation}{Total\;nitrogen\;supply}$$

$$NUtE\;=\;\frac{Leaf\;dry\;weight}{Plant\;nitrogen\;accumulation}$$

$$aNUE\;=\;\frac{LDW_{fertilized}-LDW_{not\;fertilized}}{Nitrogen\;supply\;of\;fertilizer}$$

Where LDW_fertilized_ refers to the leaf dry weight in nitrogen−treated plots, LDW_not fertilized_ refers to the leaf dry weight in non−nitrogen−treated plots.

#### 2.3.2 Root characteristics and sampling

During the maturity stage of flue−cured tobacco, root samples were collected to determine root dry matter and morphological traits. Nitrogen utilization parameters were assessed concurrently. From each plot, we randomly selected three plants and collected their entire root systems, including the crown with adhering soil. These were then transferred to a root-washing basin. Soil was gently removed by rinsing with a low−pressure stream of water until root surfaces were clean. The roots were then carefully separated by hand.

After washing, roots were spread in a single layer on the transparent tray and scanned with a color flatbed scanner (MRS−9600TFU2L; Microtek International Inc., Hsinchu, Taiwan) at 600 dpi. The images were saved in JPEG format (.jpg). They were then analyzed with WinRHIZO Pro 2007 (Regent Instruments Inc., Quebec City, QC, Canada) to obtain root morphological parameters, including total root length, root surface area and root volume. After drying to constant weight, root dry matter was determined.

Fresh root systems from three randomly selected plants in each experimental plot were collected during the root−elongation, vigorous−growth and maturity stages of flue−cured tobacco. They were immediately frozen in liquid nitrogen for subsequent analysis. Root vigor was assessed using the 2,3,5−triphenyltetrazolium chloride (TTC) reduction method (Zhao et al., 2023). In aqueous solution, TTC was colorless. Root dehydrogenases, primarily succinate dehydrogenase, reduced it to red formazan. The activity of these dehydrogenases was used as an indicator of root vigor. Root vigor was quantified as the amount of TTC reduced per unit root fresh weight during a specified incubation period (μg formazan g^−1^ FW h^−1^).

#### 2.3.3 Xylem bleeding sap collection and analysis

Collection of xylem bleeding sap from flue−cured tobacco plants was performed following Zhang et al. (2021). During the vigorous−growth stage, three randomly selected tobacco plants per plot were sampled. At 09:00 h, the stem was cut approximately 10 cm above the soil surface, and xylem bleeding sap was collected for 24 h. After the 24−h collection period, the volume of bleeding sap was measured (ml) and samples were filtered through a 0.45 μm syringe filter. The filtered samples were immediately frozen and stored at −80 °C until determination of nitrate (NO_3_^−^) concentration.

The concentration of NO_3_^−^ was quantified by the colorimetric salicylic acid–sulfuric acid method described by Zhao et al. (2017). The xylem bleeding sap flow rate was determined as the volume collected per unit time (ml h^−1^) during the 24−h collection period. The NO_3_^−^ flow rate was calculated as the product of NO_3_^−^ concentration and sap flow rate (NO_3_^−^ flow rate = NO_3_^−^ concentration × sap flow rate). It was expressed in appropriate units (μg NO_3_^−^ h^−1^ plant^−1^).

#### 2.3.4 Activity of nitrate reductase and nitrite reductase in leaf tissue

During the root elongation, vigorous growth and maturity stages of flue−cured tobacco, three plants per experimental plot were randomly selected. The fourth fully expanded leaf from the top of each plant was harvested. The harvested leaves were immediately frozen in liquid nitrogen, transported to the laboratory, and stored at −80 °C until analysis. Nitrate reductase (NR) activity was assayed *in vitro* following the protocol of Durand et al. (2003), and nitrite reductase (NiR) activity was determined according to Rao et al. (1981).

#### 2.3.5 Net photosynthetic rate and leaf nitrogen concentration

During the root−elongation, vigorous−growth and maturity stages of flue−cured tobacco, three plants per experimental plot were randomly selected. The fourth fully expanded leaf from the top of each plant was harvested. The net photosynthetic rate (*Pn*) of these leaves was measured between 09:00 and 11:00 h using a TARGAS−1 portable photosynthesis system (PP Systems, Amesbury, MA, USA). After measurement, the sampled leaves were transported to the laboratory, subjected to a kill−green treatment in an oven at 105 °C for 30 min, and then oven−dried at 80 °C to constant weight. The dried leaves were weighed to determine dry weight, ground to a fine powder, and sieved through an 80−mesh sieve to obtain samples for nitrogen analysis. Leaf nitrogen concentration was determined by the Kjeldahl method using a Kjeltec™ 8400 analyzer (Foss Analytical A/S, Hillerød, Denmark) (Bremner and Tabatabai, 2008).

#### 2.3.6 Chlorophyll fluorescence parameters analysis

During the root−elongation, vigorous−growth and maturity stages of flue−cured tobacco, three plants per experimental plot were randomly selected. The fourth fully expanded leaf from the top of each plant was sampled. Chlorophyll fluorescence parameters were measured on clear days between 09:00 and 11:00 h using an FMS2 pulse−modulation fluorometer (Hansatech Instruments Ltd., King’s Lynn, Norfolk, UK) in accordance with Maxwell and Johnson (2000). The effective quantum yield of PSII (ΦPSII) was determined on light−adapted leaves. After measuring ΦPSII, the measured area was covered with a dark−adaptation clip and shaded for 30 min to assess the maximum photochemical efficiency of PSII (*Fv/Fm*) under dark−adapted conditions.

#### 2.3.7 Respiration and total soluble sugar concentration

After topping the flue−cured tobacco plants, three plants per experimental plot were randomly selected. The fourth fully expanded leaf from the top of each plant was sampled. On clear days between 09:00 and 11:00 h, photosynthetic light−response curves of the sampled leaves were measured using a TARGAS−1 portable photosynthesis system (PP Systems, Amesbury, MA, USA). Photosynthetic photon flux density levels were manually set to 2000, 1800, 1600, 1400, 1200, 1000, 800, 500, 300, 200, 100, 50 and 0 μmol photons m^−2^ s^−1^, and the steps were applied in descending order. At each photosynthetic photon flux density level, net photosynthetic rate was recorded after the signal reached steady state. The steady−state values were then used to construct the light−response curve. The daytime dark respiration rate (*R_d, day_*) was calculated following Lv et al. (2020).

Subsequently, following Peng et al. (2013), the net photosynthetic rate (*Pn*) of the same leaves was measured at night (19:00, 21:00, 23:00, 01:00, 03:00 and 05:00) using the open gas-exchange chamber and infrared gas analyzer of a TARGAS-1 portable photosynthesis system (PP Systems, Amesbury, MA, USA). At each time point, *Pn* was recorded after the signal reached steady state, and the absolute value of dark *Pn* was taken as the nighttime dark respiration rate (*R_d, night_*). After completion of the nighttime measurements, leaves were sampled, immediately frozen in liquid nitrogen, transported to the laboratory on dry ice, and stored at −80 °C until analysis. Soluble total sugars were determined on fresh leaf tissue by the anthrone method (Wang et al., 2025).

#### 2.3.8 C/N ratio and *R_d, day_*/*P_gross_*

Field sampling and sample pretreatment were described in Section 2.3.5. For analysis, 0.20 g dry weight of the prepared sample was extracted twice with 80% ethanol at an extraction ratio of 1:50 (m/v) in an 80 °C water bath for 30 min each. After centrifugation and filtration, the supernatants were combined as the soluble sugar extract. The pellet and filter residue were retained for starch determination and hydrolyzed with 0.2 M H_2_SO_4_ in a boiling water bath for 30 min. The hydrolysate was cooled, neutralized, and filtered. The resulting supernatant was used as the starch extract. Soluble total sugars and starch were both quantified using the anthrone method (Wang et al., 2025). Specifically, 0.20 ml of each extract was mixed with anthrone–sulfuric acid reagent, heated in a boiling water bath for 10 min for color development, cooled to room temperature, and the absorbance was measured at 630 nm. Sugar contents were calculated from a glucose standard curve and converted according to sample mass and dilution factors. The corresponding nitrogen content was determined and described in Section 2.3.5. C/N ratio = (soluble sugar content + starch content)/total nitrogen content (Feng et al., 2024a).

The photosynthetic light−response curve is described in Section 2.3.7 above. The respiration fraction of flue-cured tobacco (*R_d, day_*/*P_gross_*), calculated from this curve, reflects the instantaneous carbon balance of the leaf. Here, *R_d, day_* denotes daytime dark respiration rate (μmol CO_2_ m^−2^ s^−1^), and *P_gross_* denotes the gross photosynthetic rate (μmol CO_2_ m^−2^ s^−1^) at a standardized light intensity of 1000 μmol photons m^−2^ s^−1^. *R_d, day_*/*P_gross_* represents the proportion of daytime dark respiration to gross photosynthesis (Qie et al., 2023).

### 2.4 Statistical analyses

Statistical analyses were performed using IBM SPSS Statistics 20.0 (IBM Corp., Armonk, NY, USA). One−way analysis of variance (ANOVA) was conducted, and post−hoc multiple comparisons were performed using Duncan’s multiple range test at *p* < 0.05. Pearson correlation coefficients were calculated to assess relationships among NUpE, NUtE, NUE and physiological indicators. Path coefficients were estimated by stepwise linear regression performed in SPSS. Data are presented as mean ± SE, and different letters indicate significant differences at *p* < 0.05. Figures were generated in Microsoft Excel 2010 (Microsoft Corp., Redmond, WA, USA) and were edited in Adobe Photoshop CC 2018 (Adobe Inc., San Jose, CA, USA) and Microsoft PowerPoint 2010 (Microsoft Corp., Redmond, WA, USA).

## 3 Results

### 3.1 Yield and nitrogen−use characteristics across flue−cured tobacco genotypes

Different flue−cured tobacco genotypes exhibited significant differences in yield and nitrogen−use characteristics (**Table 1**). Overall across N rates, Yueyan 97 had the highest yield and NUE components, K326 was intermediate, and Yunyan 87 was lowest. At the same N application rate, Yueyan 97 showed the highest yield, N utilization efficiency, and agronomic N use efficiency. Comparative analysis of N uptake efficiency revealed no significant differences between Yueyan 97 and K326, both of which had significantly higher efficiencies than Yunyan 87. In terms of N use efficiency, Yueyan 97 exhibited the best performance, followed by K326, while Yunyan 87 recorded the lowest efficiency. Overall, Yunyan 87 was lower in N uptake, utilization, and overall use efficiency. Conversely, Yueyan 97 was higher in NUpE, NUtE, and NUE. In contrast, K326 showed high NUpE but only intermediate NUE, consistent with its lower NUtE.

**Table 1:** Yield and nitrogen−use characteristics of three tobacco genotypes under varying nitrogen levels.

Year	Nitrogen level	Genotype	Yield (kg ha^-1^)	N use efficiency (kg kg^-1^)	N uptake efficiency (kg kg^-1^)	N utilization efficiency (kg kg^-1^)	Agronomic N use efficiency (kg kg^-1^)
2021	Traditional Nitrogen Application Rate	Yunyan 87	2031.44 ± 23.75b	13.01 ± 0.19c	0.43 ± 0.01b	30.16 ± 0.80b	10.18 ± 0.26c
		Yueyan 97	2634.14 ± 44.08a	20.29 ± 0.58a	0.49 ± 0.01a	41.52 ± 1.60a	16.09 ± 0.80a
		K326	2090.85 ± 127.61b	16.66 ± 0.11b	0.50 ± 0.01a	33.58 ± 1.19b	13.06 ± 0.16b
	Reduce Nitrogen Application Rate by 30%	Yunyan 87	1915.76 ± 72.17b	13.62 ± 0.41c	0.40 ± 0.01b	34.12 ± 0.32b	9.90 ± 0.62b
		Yueyan 97	2484.42 ± 50.22a	22.03 ± 0.33a	0.47 ± 0.00a	46.86 ± 0.91a	16.96 ± 0.51a
		K326	1952.09 ± 101.09b	15.69 ± 0.27b	0.48 ± 0.01a	32.79 ± 0.89b	10.02 ± 0.42b
2022	Traditional Nitrogen Application Rate	Yunyan 87	1419.99 ± 43.17b	11.64 ± 1.01b	0.35 ± 0.01b	32.74 ± 1.78b	7.00 ± 1.12b
		Yueyan 97	1977.66 ± 8.61a	18.94 ± 0.74a	0.42 ± 0.01a	45.07 ± 1.74a	12.23 ± 0.82a
		K326	1477.95 ± 20.24b	14.64 ± 0.88b	0.43 ± 0.01a	34.04 ± 2.46b	9.03 ± 0.99ab
	Reduce Nitrogen Application Rate by 30%	Yunyan 87	1342.87 ± 49.15b	12.64 ± 0.54c	0.35 ± 0.03b	36.82 ± 2.06ab	6.19 ± 0.62b
		Yueyan 97	1884.48 ± 42.87a	22.59 ± 0.38a	0.50 ± 0.03a	45.88 ± 3.96a	13.60 ± 0.44a
		K326	1354.32 ± 16.26b	14.84 ± 0.53b	0.43 ± 0.02ab	34.68 ± 2.13b	6.37 ± 0.14b

Data are means and standard error (SE) for 3 tobacco genotypes under two nitrogen levels in 2021 and 2022. Value are Mean ± SE (n = 3). Values followed by different letters indicate significant differences within the same nitrogen level in each year (p < 0.05, ANOVA−Duncan’s multiple range test).

### 3.2 Root morphology and vigor dynamics across flue−cured tobacco genotypes

**Figure 2:**
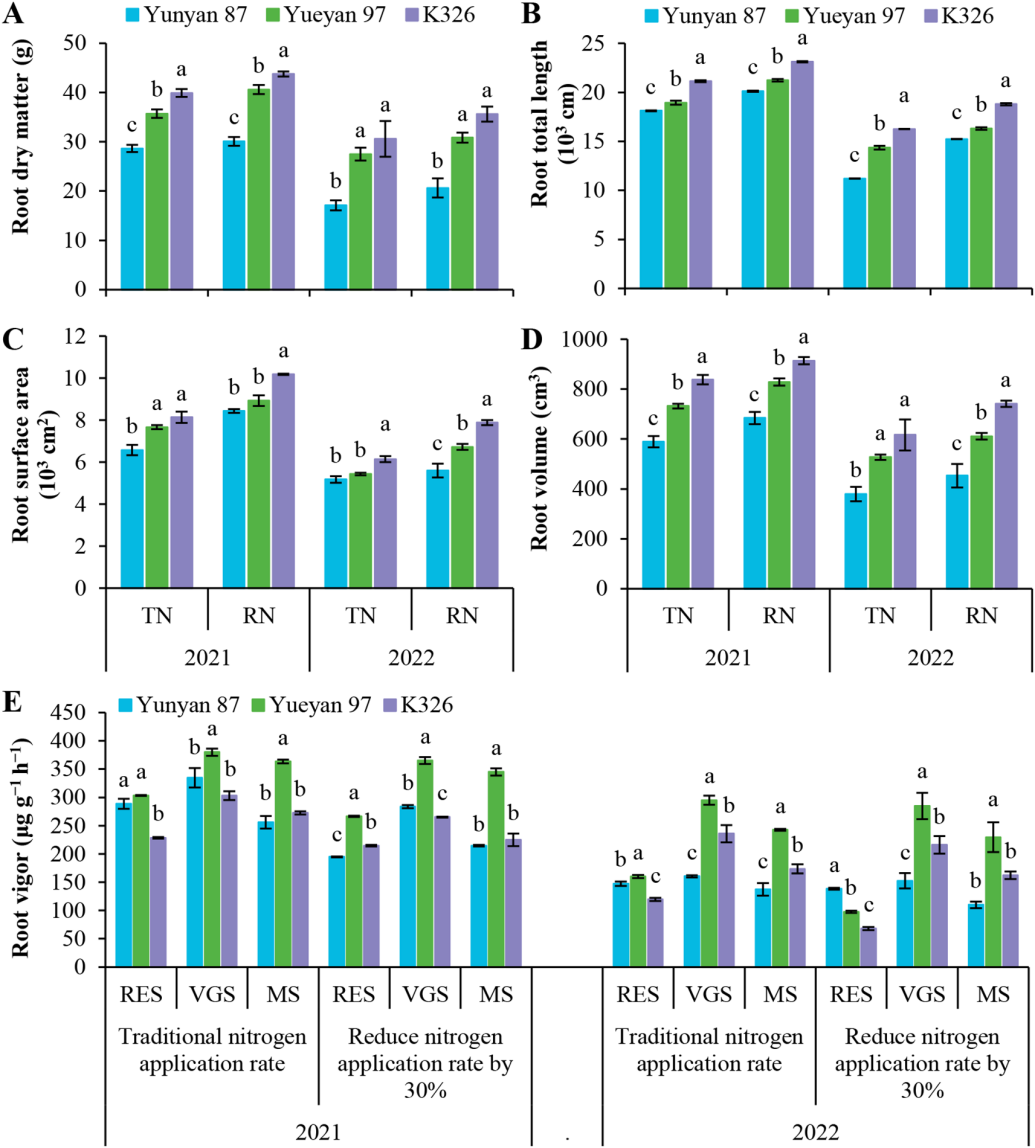
Variations in root indices among three flue−cured tobacco genotypes under different nitrogen levels. **(A)** Root dry matter at maturity stage in 2021 and 2022. **(B)** Root total length at maturity stage in 2021 and 2022. **(C)** Root surface area at maturity stage in 2021 and 2022. **(D)** Root volume at maturity stage in 2021 and 2022. **(E)** Root vigor at different growth stages in 2021 and 2022. Error bars represent standard errors. Different lowercase letters indicate statistically significant differences between tobacco genotypes (*p* < 0.05, ANOVA−Duncan’s multiple range test). TN, traditional nitrogen application rate; RN, reduce nitrogen application rate by 30%; RES, root−elongation stage; VGS, vigorous−growth stage; MS, maturity stage.

To investigate the factors underlying genotypic differences in NUpE, root dry matter and morphology were measured at maturity. Root vigor was assessed at multiple growth stages (**Figure 2**). Under identical N application rate, root dry matter, total length, surface area and volume were highest in K326, intermediate in Yueyan 97 and lowest in Yunyan 87. Yueyan 97 showed greater root vigor than Yunyan 87 and K326 at most sampling dates (*p* < 0.05). An exception occurred during the root-elongation stage in 2022 under the reduced-N application rate, when its root vigor was significantly lower than Yunyan 87 (*p* < 0.05). Comparisons of root vigor between Yunyan 87 and K326 showed no consistent pattern across years, growth stages and N treatments. Root biomass, morphology and vigor were associated with observed differences in NUpE (**Table 1**). The superior root morphology of K326 and the greater root vigor of Yueyan 97 are consistent with their respective NUpE patterns (**Table 1**).

### 3.3 Xylem bleeding sap rates and NO_3_^-^ translocation rates across flue−cured tobacco genotypes

**Figure 3:**
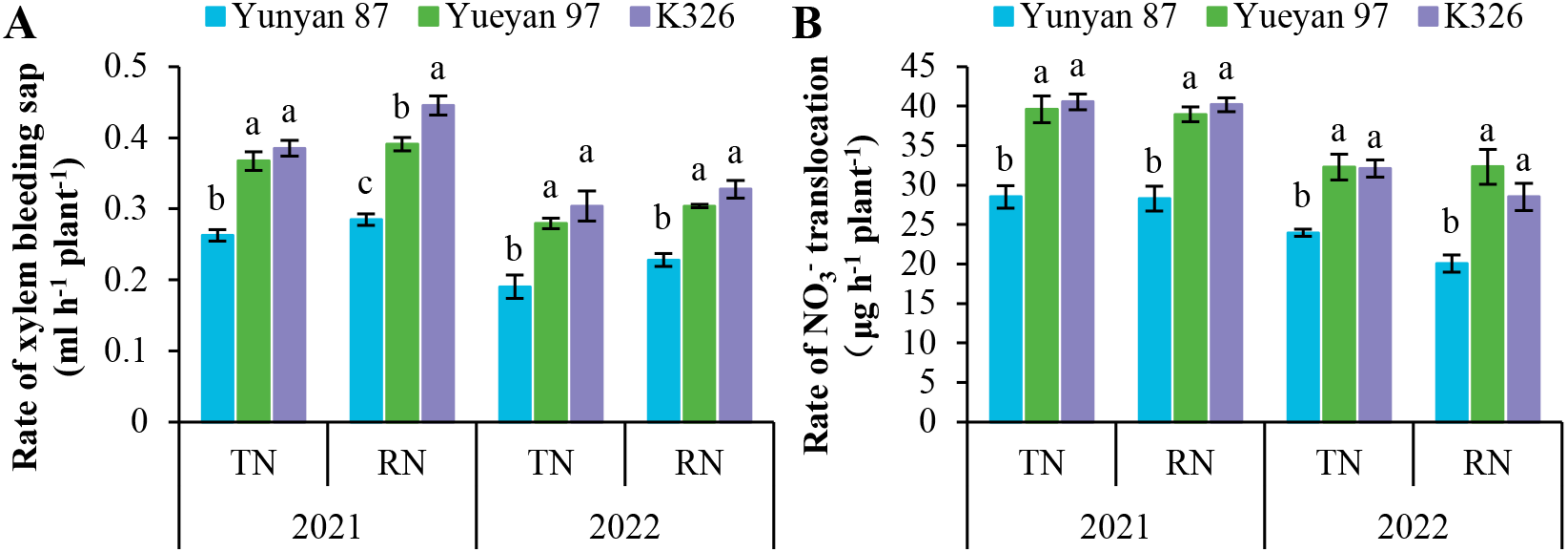
Comparison of xylem bleeding sap rates and NO_3_^-^ translocation rates among three tobacco genotypes under varying nitrogen levels. **(A)** Rate of xylem bleeding sap at vigorous− growth stage in 2021 and 2022. **(B)** NO_3_^−^ translocation rate at vigorous−growth stage in 2021 and 2022. Error bars represent standard errors. Different lowercase letters indicate statistically significant differences between tobacco genotypes (*p* < 0.05, ANOVA−Duncan’s multiple range test). TN, traditional nitrogen application rate; RN, reduce nitrogen application rate by 30%.

To investigate genotypic differences in root−to−shoot N transport capacity, xylem bleeding sap flow from stems and NO_3_^−^ translocation rates were measured (**Figure 3**). Under identical N application rates, the xylem bleeding sap rate of Yueyan 97 and K326 was significantly higher than that of Yunyan 87 (*p* < 0.05). K326 had a slightly higher mean xylem bleeding sap rate than Yueyan 97 overall. However, this difference was significant only under the reduced−N application rate in 2021 (*p* < 0.05) and not under other N treatments (*p* > 0.05). NO_3_^−^ translocation rates followed a similar pattern. Yueyan 97 and K326 showed significantly higher NO_3_^−^ translocation rates than Yunyan 87 (*p* < 0.05), while no significant differences were observed between Yueyan 97 and K326 across most treatments (*p* > 0.05). These findings are consistent with the higher N uptake and accumulation observed in Yueyan 97 and K326 (**Table 1**).

### 3.4 Nitrate reductase and nitrite reductase activities across flue−cured tobacco genotypes

**Figure 4:**
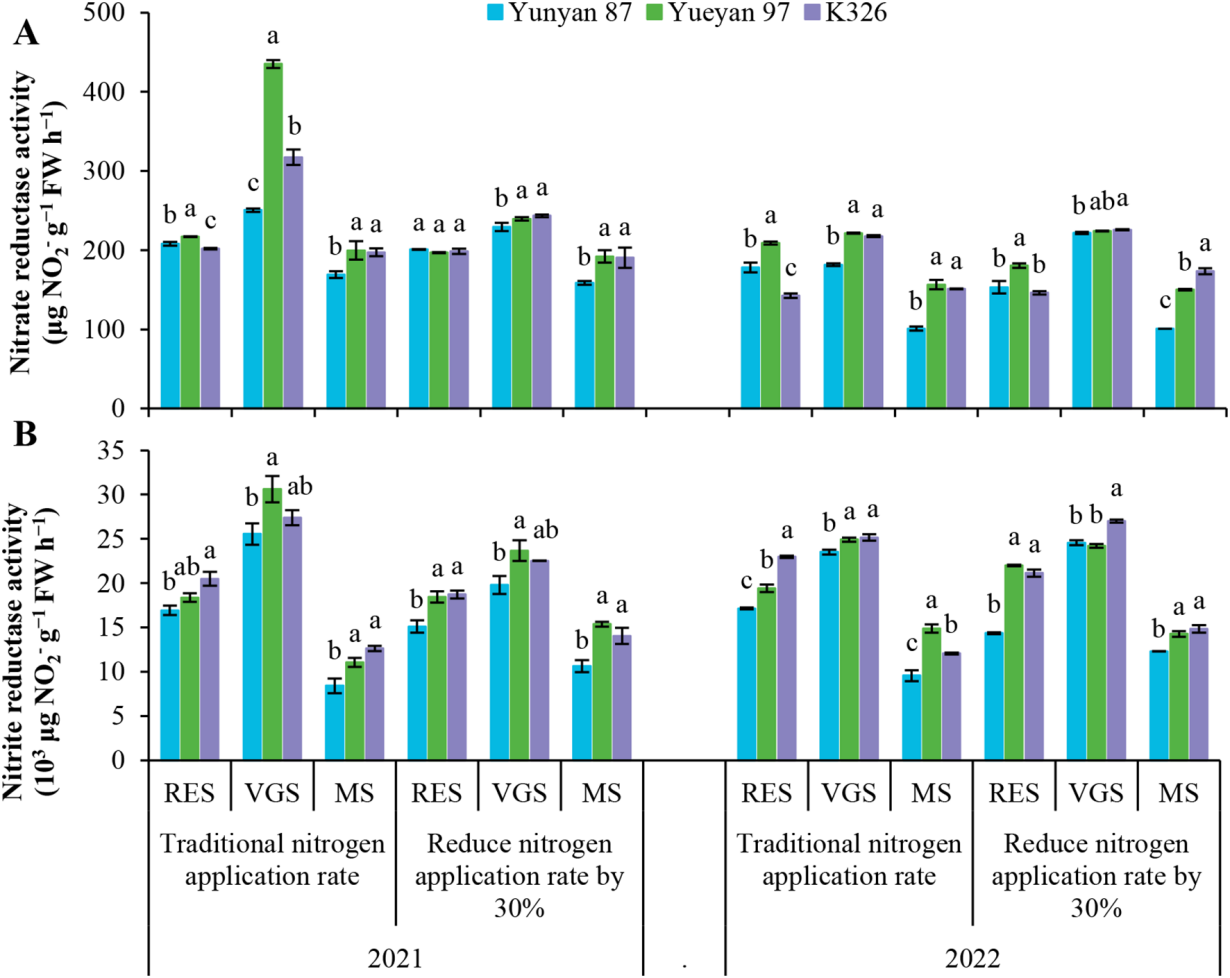
Variation in nitrate reductase and nitrite reductase activities in leaf tissues of three tobacco genotypes under different nitrogen levels. **(A)** Nitrate reductase activity at different growth stages in 2021 and 2022. **(B)** Nitrite reductase activity at different growth stages in 2021 and 2022. Error bars represent standard errors. Different lowercase letters indicate statistically significant differences between tobacco genotypes (*p* < 0.05, ANOVA−Duncan’s multiple range test). RES, root−elongation stage; VGS, vigorous−growth stage; MS, maturity stage.

To investigate genotype−dependent differences in N assimilation, NR and NiR activities in leaves were measured at multiple developmental stages under two N fertilizer application rates (**Figure 4**). At the root−elongation and vigorous−growth stages, NR activity was significantly higher in Yueyan 97 and K326 than in Yunyan 87 under both N application rates. In 2021 under the traditional N application rate, NR activity in Yueyan 97 was significantly higher than in K326 at the root−elongation and vigorous−growth stages (*p* < 0.05). However, this advantage was not observed under reduced−N application rate. Overall, NR activities were higher in Yueyan 97 and K326 than in Yunyan 87 across N application rates. Yueyan 97 exceeded K326 under the traditional N application rate in 2021. NiR activity was higher in Yueyan 97 and K326 than in Yunyan 87 across most sampling dates (*p* < 0.05). A significant difference in NiR activity between Yueyan 97 and K326 was observed only in 2022. No significant difference was found between them in 2021 under either N application rate (*p* > 0.05).

These enzyme activity patterns were consistent with the higher N uptake and accumulation observed for Yueyan 97 and K326 (**Table 1**). Differences between Yueyan 97 and K326 in NR and NiR activities under the two N application rates were generally small.

### 3.5 Net photosynthetic rate and leaf N concentration across flue−cured tobacco genotypes

**Figure 5:**
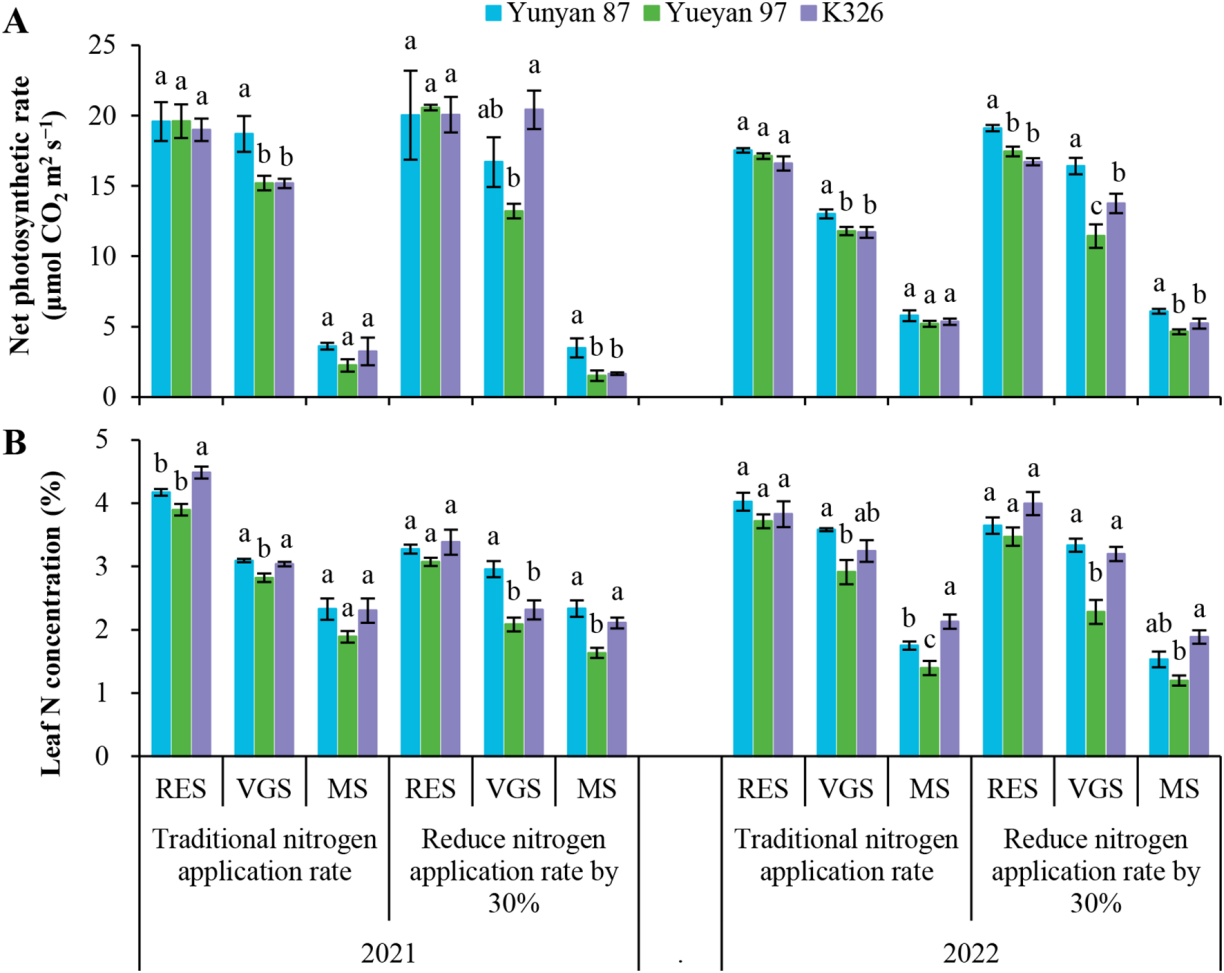
Comparison of net photosynthetic rate and leaf N concentration among three tobacco genotypes under varying nitrogen levels. **(A)** Net photosynthetic rate at different growth stages in 2021 and 2022. **(B)** Leaf N concentration at different growth stages in 2021 and 2022. Error bars represent standard errors. Different lowercase letters indicate statistically significant differences between tobacco genotypes (*p* < 0.05, ANOVA−Duncan’s multiple range test). RES, root−elongation stage; VGS, vigorous−growth stage; MS, maturity stage.

To investigate genotype−dependent differences in photosynthetic regulation and leaf N status, net photosynthetic rate and leaf N concentration were measured at multiple growth stages (**Figure 5**). Under the traditional N application rate, no significant differences in net photosynthetic rate were detected between Yueyan 97 and Yunyan 87 at the root−elongation and maturity stages (*p* > 0.05). However, during the vigorous−growth stage, Yueyan 97 had a significantly lower net photosynthetic rate than Yunyan 87, decreasing by 18.72% in 2021 and 10.25% in 2022 (*p* < 0.05). Leaf N concentration in Yueyan 97 at the vigorous−growth stage was also lower than in Yunyan 87 (*p* < 0.05). Similarly, under the traditional N application rate, K326 exhibited a significantly lower net photosynthetic rate than Yunyan 87 during the vigorous−growth stage (*p* < 0.05). In contrast, leaf N concentration did not differ significantly between K326 and Yunyan 87 at that stage (*p* > 0.05).

Under the reduced−N application rate, the net photosynthetic rate of Yueyan 97 was lower than that of Yunyan 87, with the difference being significant at the maturity and vigorous−growth stages in 2022 (*p* < 0.05). Leaf N concentration in Yueyan 97 at those stages in 2022 was also reduced relative to Yunyan 87. For K326, the mean net photosynthetic rate across sampling dates was lower than that of Yunyan 87. However, under the reduced−N application rate, K326 exhibited a significantly higher net photosynthetic rate than Yueyan 97 during the vigorous−growth stage (*p* < 0.05). At that stage, leaf N concentration did not differ significantly between K326 and Yunyan 87.

Across N application rates, Yunyan 87 often had higher net photosynthetic rates than Yueyan 97 and K326, and Yueyan 97 tended to be the lowest. In the later growth stages, leaf N concentrations did not differ significantly between Yunyan 87 and K326, and both were significantly higher than that of Yueyan 97.

### 3.6 Comparison of chlorophyll fluorescence parameters across flue−cured tobacco genotypes

**Figure 6:**
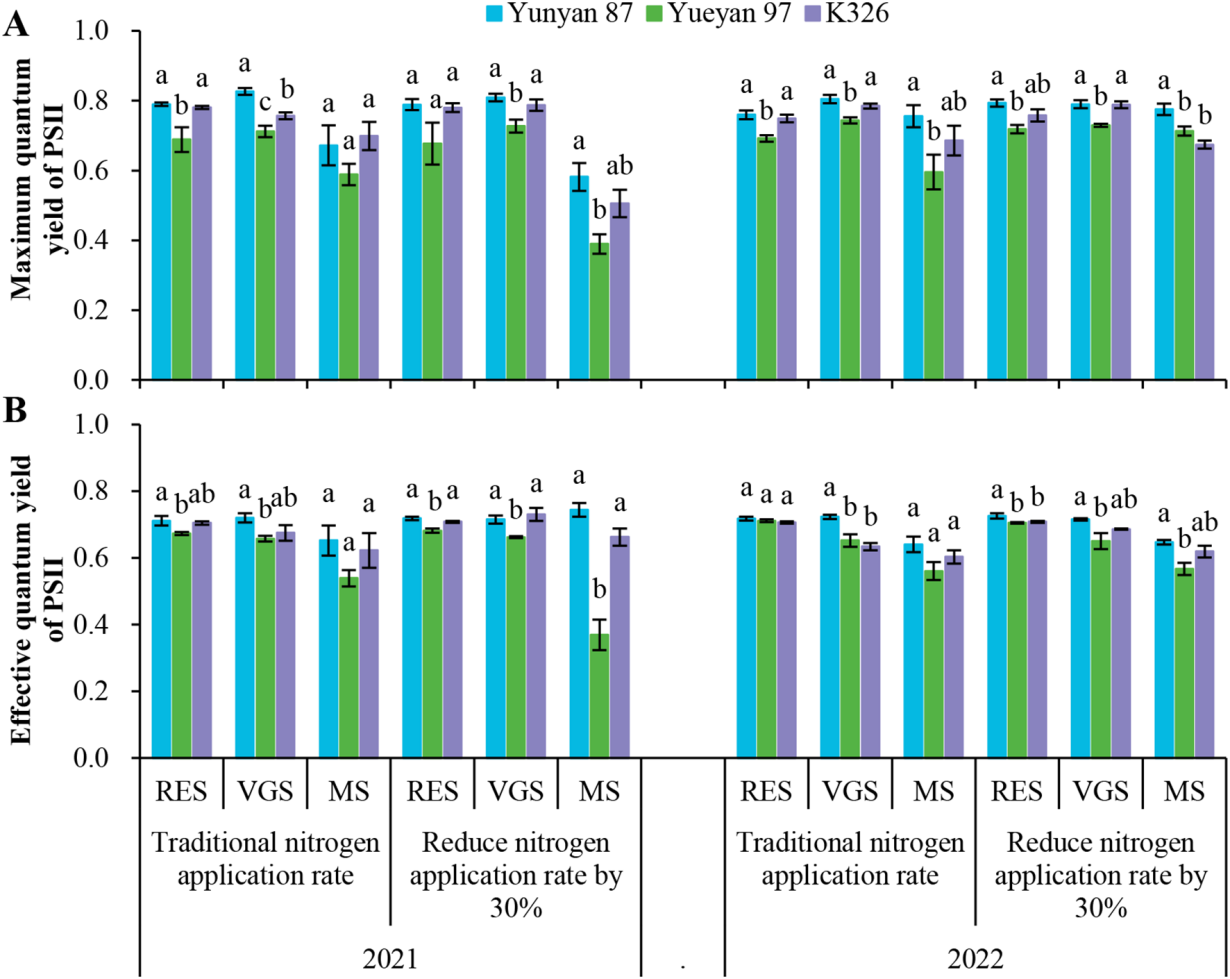
Variation in chlorophyll fluorescence parameters among three tobacco genotypes under different nitrogen levels. **(A)** Maximum quantum yield of PSII at different growth stages in 2021 and 2022. **(B)** Effective quantum yield of PSII at different growth stages in 2021 and 2022. Error bars represent standard errors. Different lowercase letters indicate statistically significant differences between tobacco genotypes (*p* < 0.05, ANOVA−Duncan’s multiple range test). PSII, photosystem II; RES, root−elongation stage; VGS, vigorous−growth stage; MS, maturity stage.

Analysis of the data in **Table 1** and **Figure 5** revealed an unexpected relationship between net photosynthetic rate and NUE. Yunyan 87 exhibited the highest net photosynthetic rate but the lowest NUE, whereas Yueyan 97 showed the lowest net photosynthetic rate but the highest NUE. To investigate the physiological basis of these observations, chlorophyll fluorescence parameters *Fv/Fm* and ΦPSII were measured in leaves at the root−elongation, vigorous−growth and maturity stages (**Figure 6**).

Under the traditional N application rate, *Fv/Fm* of Yueyan 97 was significantly lower than that of Yunyan 87 at multiple time points (all *p* < 0.05). K326 showed a significantly lower *Fv/Fm* than Yunyan 87 only during the vigorous-growth stage in 2021. No significant differences were observed at other sampling times (*p* > 0.05). Under the reduced-N application rate, the pattern of *Fv/Fm* for Yueyan 97 was similar to that under the traditional N application rate. Although *Fv/Fm* values for K326 were lower than those for Yunyan 87 during the maturity stage in 2022, the differences were not statistically significant at other sampling times (*p* > 0.05). For ΦPSII, Yueyan 97 was lower than Yunyan 87 at all sampling times under reduced-N (all *p* < 0.05), and it also differed at several times under traditional N. K326 differed from Yunyan 87 only sporadically.

These fluorescence results were consistent with the lower net photosynthetic rate observed for Yueyan 97 relative to Yunyan 87 (**Figure 5**). The physiological mechanisms underlying the higher nitrogen use efficiency of Yueyan 97 were not established in this study and require further investigation.

### 3.7 Respiration and total soluble sugar concentration across flue−cured tobacco genotypes

**Figure 7:**
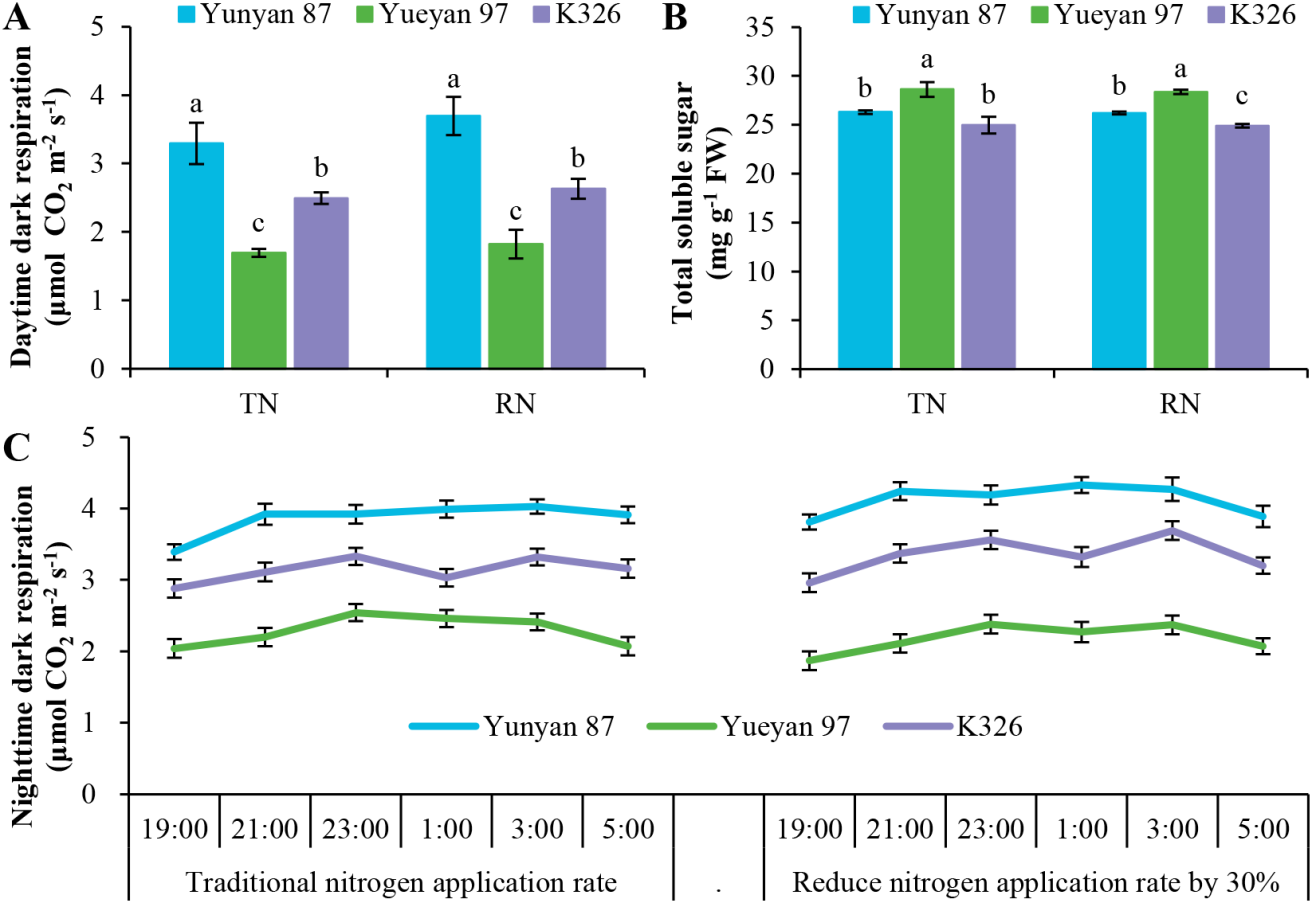
Variations in respiration rate and total soluble sugar concentration among three tobacco genotypes at maturity stage in 2022. **(A)** Daytime dark respiration. **(B)** Total soluble sugar concentration in fresh leaves. **(C)** Nighttime dark respiration at 19: 00, 21:00 and 23:00 on the first day and at 1:00, 3:00 and 5:00 in the morning of the next day. Error bars represent standard errors. Different lowercase letters indicate statistically significant differences between tobacco genotypes in **(A, B)** (*p* < 0.05, ANOVA−Duncan’s multiple range test). TN, traditional nitrogen application rate; RN, reduce nitrogen application rate by 30%.

To investigate why Yueyan 97 showed higher yield and nitrogen use efficiency despite a markedly lower net photosynthetic rate, *R_d, day_*, *R_d, night_* and leaf total soluble sugar concentration were measured for the three tobacco genotypes during the maturity stage in 2022 (**Figure 7**). Overall, dark respiration rates under both daytime and nighttime conditions differed significantly among the three genotypes (*p* < 0.05; **Figures 7A, C**), with Yueyan 97 lowest, K326 intermediate, and Yunyan 87 highest. Specifically, relative to Yunyan 87, *R_d, day_* of Yueyan 97 was reduced by 48.63% under the traditional N application rate and by 50.68% under the reduced−N application rate (both *p* < 0.05; **Figure 7A**). By contrast, *R_d, day_* of K326 was reduced by 24.32% and 28.73% relative to Yunyan 87 under the traditional and reduced−N application rates, respectively (both *p* < 0.05; **Figure 7A**). *R_d, night_* was relatively stable throughout the night from 19:00 to 05:00 the next day. Under the traditional N application rate, *R_d, night_* of Yunyan 87, Yueyan 97 and K326 ranged from 3.39–4.03, 2.04–2.54 and 2.88–3.33 μmol CO_2_ m^−2^ s^−1^, respectively (overall *p* < 0.05; **Figure 7C**). Under the reduced−N application rate, their *R_d, night_* ranged from 3.81–4.33, 1.87–2.38 and 2.96–3.69 μmol CO_2_ m^−2^ s^−1^, respectively (overall *p* < 0.05; **Figure 7C**). Leaf total soluble sugar concentrations in Yueyan 97 were significantly higher than those in Yunyan 87 and K326 under both N application rates (*p* < 0.05; **Figure 7B**). When Yunyan 87 and K326 were compared directly, a significant difference in soluble sugar was detected only under the reduced−N application rate (*p* < 0.05). No significant difference was found under the traditional N application rate (*p* > 0.05). Integrated analysis showed that reduced respiratory carbon loss in Yueyan 97 likely contributes to its higher nitrogen use efficiency and yield. A detailed interpretation is provided in the Discussion.

### 3.8 Variations in C/N ratio and *R_d, day_*/*P_gross_* among flue−cured tobacco genotypes

**Figure 8:**
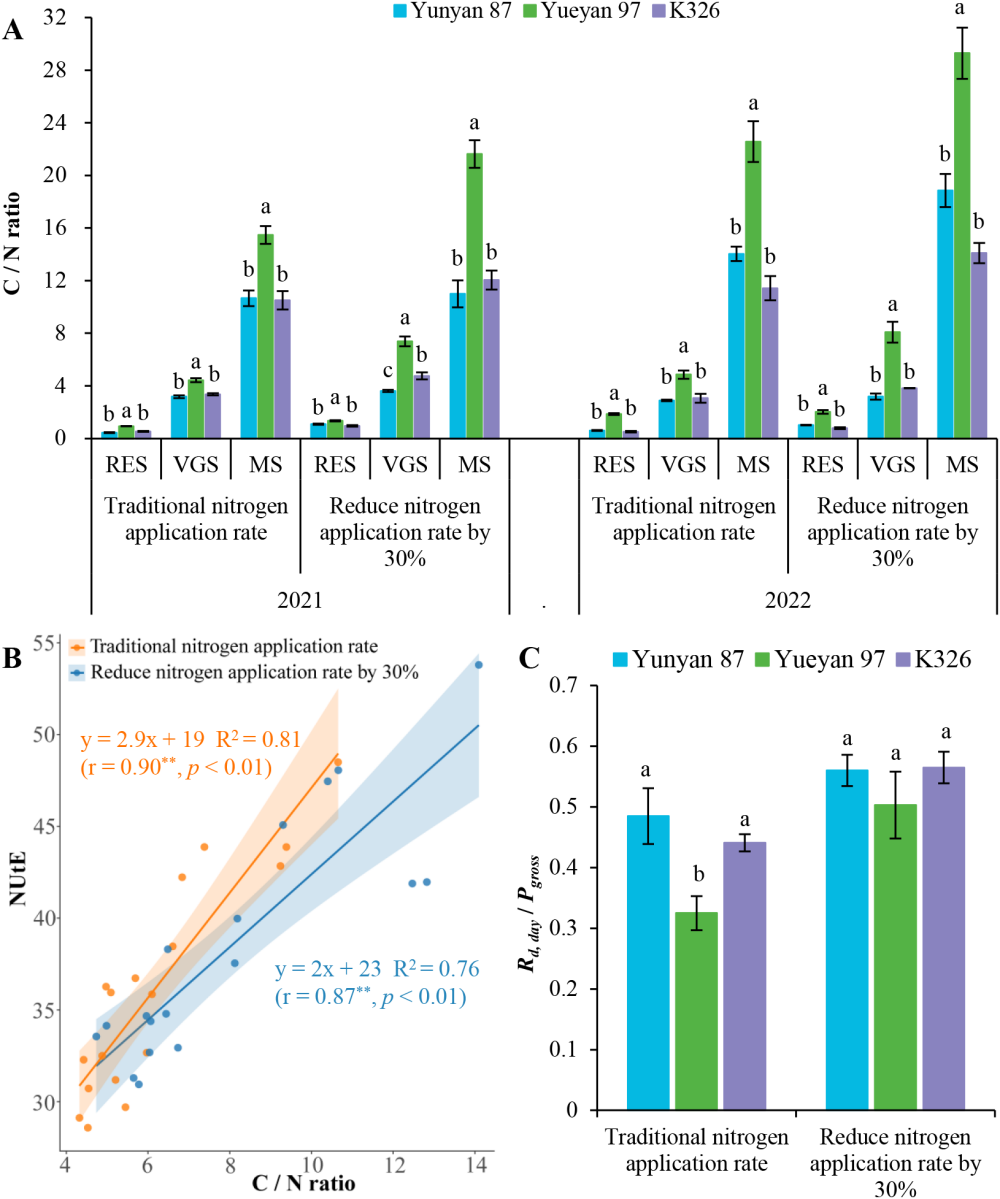
Variations in C/N ratio and *R_d, day_*/*P_gross_* among three tobacco genotypes. **(A)** C/N ratio at different growth stages in 2021 and 2022. C/N ratio = (soluble sugar content + starch content)/total nitrogen content. RES: root−elongation stage; VGS: vigorous−growth stage; MS: maturity stage. **(B)** Correlation between C/N ratio and NUtE (with linear fit and 95% CI). In panel **(B)**, r denotes the Pearson correlation coefficient and *p* the two-tailed significance; ** indicates *p* < 0.01. **(C)***R_d, day_*/*P_gross_* at maturity stage in 2022. *R_d, day_*: daytime dark respiration; *P_gross_*: gross photosynthetic rate. Error bars represent standard errors. Different lowercase letters indicate statistically significant differences between tobacco genotypes in **(A, C)** (*p* < 0.05, ANOVA−Duncan’s multiple range test).

To further elucidate the physiological context of “lower photosynthesis yet higher NUtE,” we compared leaf C/N, NUtE, and the proportion of daytime dark respiration in gross photosynthesis (*R_d, day_*/*P_gross_*) across genotypes (**Figure 8**). Under both nitrogen application rates, Yueyan 97 showed significantly higher leaf C/N than Yunyan 87 and K326 (*p* < 0.05), whereas Yunyan 87 and K326 did not differ significantly (*p* > 0.05). Correlation analysis indicated a significant positive association between NUtE and C/N, suggesting that higher leaf C/N tends to accompany higher NUtE.

Regarding respiratory costs, Yueyan 97 had consistently lower *R_d, day_*/*P_gross_* than Yunyan 87 and K326, with the difference reaching significance under the traditional nitrogen application rate (*p* < 0.05). In contrast, Yunyan 87 and K326 were similar. This pattern is consistent with a lower relative dark- respiration burden in Yueyan 97. In other words, a smaller fraction of assimilated carbon seems to be respired per unit time.

### 3.9 Correlation and path coefficient analysis of physiological indexes, NUpE, NUtE and NUE among flue−cured tobacco genotypes

**Figure 9:**
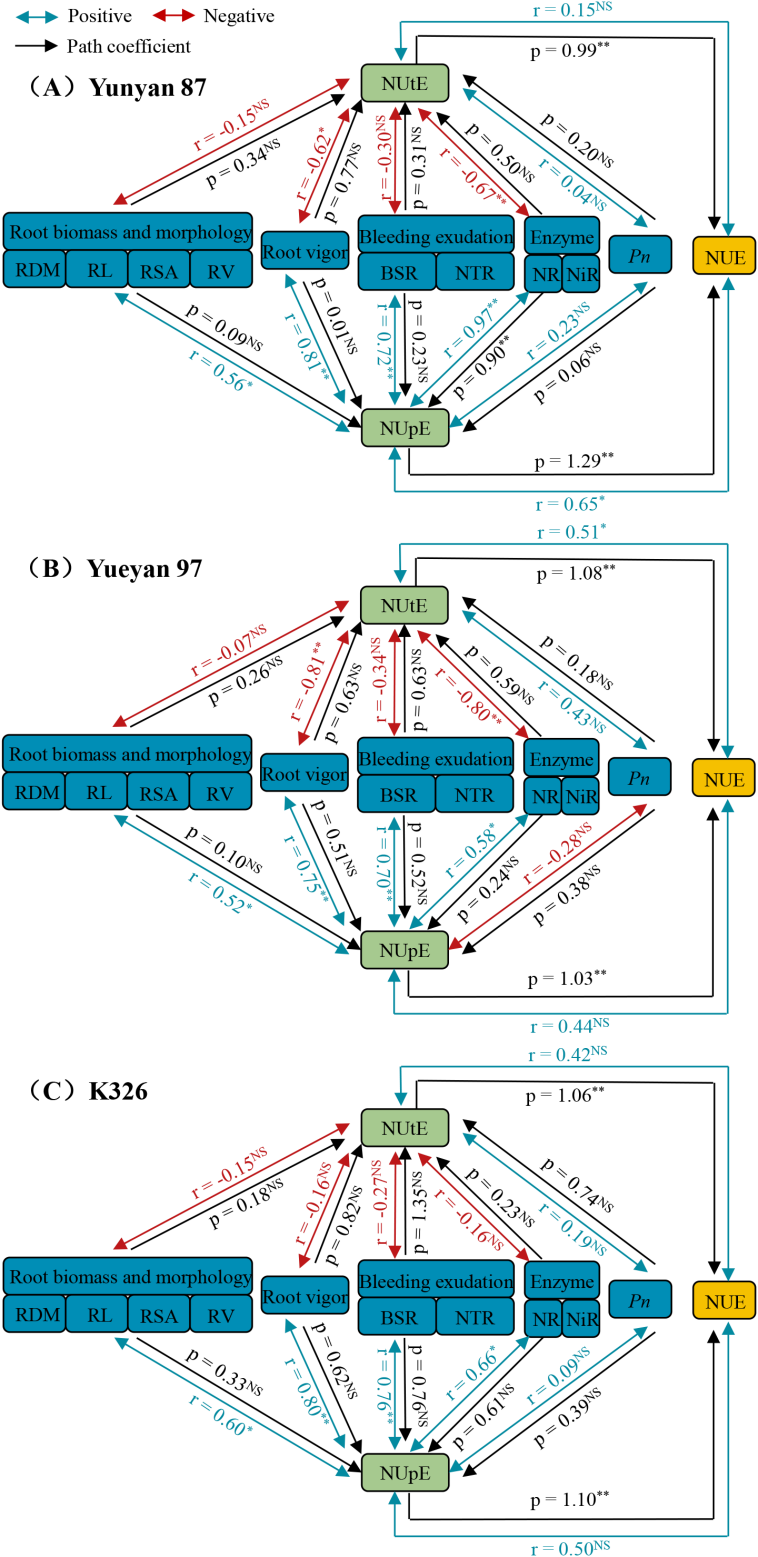
Correlation and path coefficients of physiological indexes, NUpE, NUtE and NUE among Yunyan 87 **(A)**, Yueyan 97 **(B)** and K326 **(C)**. Double arrowhead lines with r indicate correlativity. The blue and red lines represent positive and negative effects, respectively. Single arrowed lines with p denote direct path coefficient. Result is the average from 2021 to 2022. NS, * and ** indicate no significant, significant differences at 0.05 and 0.01 levels. RDM, root dry matter; RL, root total length; RSA, root surface area; RV, root volume; BSR, rate of xylem bleeding sap; NTR, rate of NO_3_^−^ translocation; NR, nitrate reductase activity; NiR, nitrite reductase activity; *Pn*, net photosynthetic rate; NUpE, nitrogen uptake efficiency; NUtE, nitrogen utilization efficiency; NUE, nitrogen use efficiency.

Correlation and path coefficient analyses were performed to assess NUpE, NUtE and NUE across flue−cured tobacco genotypes, with particular focus on root biomass and morphology, root vigor, bleeding exudation, leaf enzyme activities, and net photosynthetic rate (**Figure 9**). In this path analysis, the coefficients quantify associations defined by the model. On their own, they do not establish causality. We report them to indicate the relative strength and direction of relationships after accounting for correlations among predictors. Correlation analysis showed that root biomass, root morphology, root vigor, bleeding exudation and leaf enzyme activities were positively and significantly correlated with NUpE in all three genotypes (*p* < 0.05 or *p* < 0.01). In contrast, net photosynthetic rate was not significantly correlated with NUpE (*p* > 0.05). These variables therefore were the primary factors associated with nitrogen uptake in tobacco.

In the analysis of NUtE, NUtE was negatively correlated with root biomass, root morphology, root vigor, bleeding exudation and leaf enzyme activities. Significant correlations between these variables and NUtE were detected in Yunyan 87 and Yueyan 97 but not in K326. These results may suggest that enhancing NUpE in tobacco could lead to excessive nitrogen accumulation within the plant, potentially resulting in a decline in NUtE. Furthermore, a positive but non−significant correlation between NUtE and net photosynthetic rate was observed. Upon considering the yield and nitrogen efficiency of each tobacco genotype (**Table 1**), alongside the C/N ratio and *R_d, day_*/*P_gross_* (**Figure 8**), it can be inferred that the increased NUtE observed in tobacco may be more closely associated with a reduction in respiratory consumption.

For Yunyan 87, the standardized direct path coefficients of NUpE and NUtE to NUE were 1.29 and 0.99, respectively. Both coefficients were statistically significant. A significant positive correlation between NUpE and NUE was observed for this genotype, whereas the correlation between NUtE and NUE was not significant. These results suggested that NUpE contributed more to NUE than did NUtE in Yunyan 87. In Yueyan 97 the direct path coefficients of NUpE and NUtE to NUE were 1.03 and 1.08, and both were significant. However, the correlation between NUpE and NUE was not significant, whereas NUtE showed a significant positive correlation with NUE. This pattern was consistent with a greater contribution of NUtE than NUpE to NUE in Yueyan 97. For K326, the direct path coefficients of NUpE and NUtE to NUE were 1.10 and 1.06, and both were significant. However, the correlations of NUpE and NUtE with NUE were not significant. These results were consistent with a slightly higher direct contribution of NUpE than NUtE to NUE in K326. However, the difference between the two contributions was small. These model-based direct and indirect coefficients reflect associations within the specified path structure. They should not be interpreted as causal effects.

**Figure 10:**
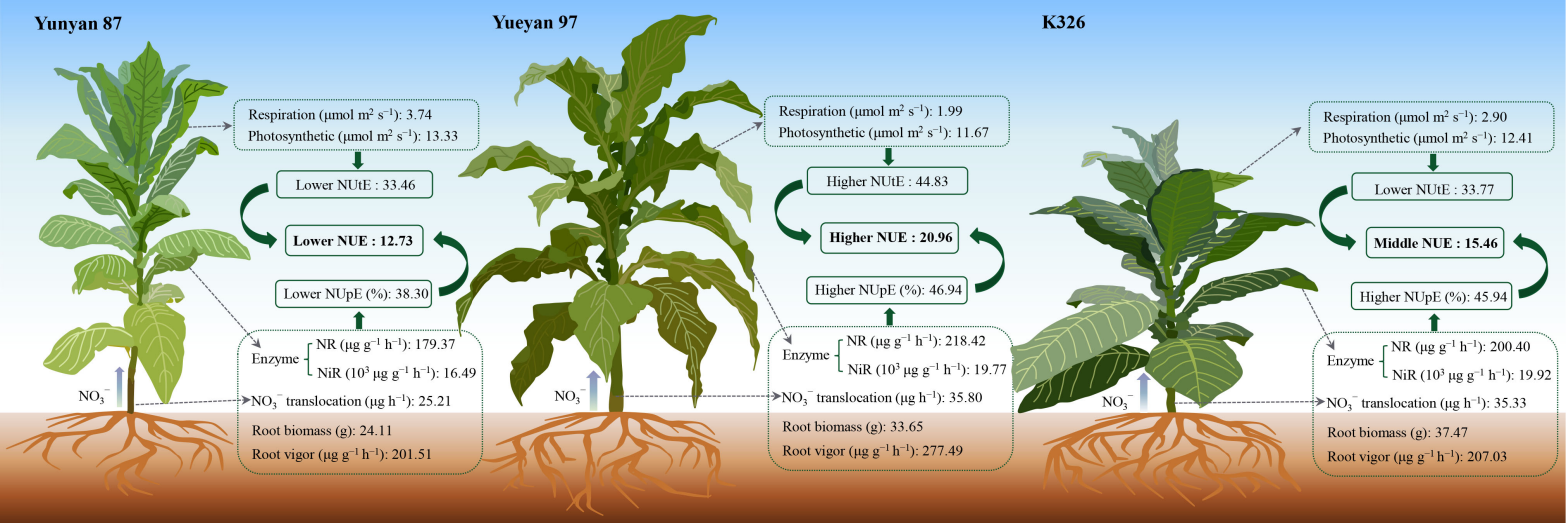
Comparative summary of NUE, NUpE, NUtE and their influencing factors among Yunyan 87, Yueyan 97 and K326. The value of respiration is the average of the results in 2022, and the values of other indicators are the average of two years in 2021 and 2022. NUtE, nitrogen utilization efficiency; NUE, nitrogen use efficiency; NUpE, nitrogen uptake efficiency; NR, nitrate reductase activity; NiR, nitrite reductase activity.

Overall, root biomass and morphology, root vigor, bleeding exudation and leaf enzyme activity were identified as the main variables associated with NUpE in Yunyan 87, Yueyan 97 and K326. This was indicated by positive correlations (**Figures 9**, **10**). An increase in NUtE was associated with lower respiration rates in the examined genotypes (**Figures 5**, **7**). Comparison of the standardized direct path coefficients to NUE among genotypes revealed genotype−specific patterns. NUpE contributed more than NUtE in Yunyan 87, NUtE contributed more in Yueyan 97, and in K326 the two contributions were numerically close. Even so, the path coefficients and associated correlations are informative for partitioning correlations among interrelated traits. They are also useful for prioritizing testable hypotheses across genotypes.

## 4 Discussion

We compared nitrogen efficiency among three flue−cured tobacco genotypes: Yunyan 87, Yueyan 97, and K326. Yunyan 87 exhibited low NUpE, low NUtE and low NUE, whereas Yueyan 97 had high NUpE, high NUtE and high NUE. K326 showed high NUpE, low NUtE and intermediate NUE (**Table 1**). Compared with the low NUpE genotype Yunyan 87, Yueyan 97 and K326 (high NUpE genotypes) had greater root biomass and improved root morphology, including longer total root length, larger surface area, and greater volume. They also showed higher root vigor, larger bleeding-sap volume, higher NO_3_^−^ concentration in bleeding sap, and elevated NR and NiR activities (**Figures 2**-**4**). These traits were positively correlated with NUpE (**Figure 9**).

Although Yueyan 97 had a lower net photosynthetic rate than the low−NUtE genotypes, its respiration rate was significantly lower (**Figures 5**, **7**). Accordingly, higher NUtE in Yueyan 97 coincided with reduced respiratory consumption. This is consistent with the possibility that lower respiratory N and C losses could contribute to higher NUtE despite a lower gross photosynthetic rate. Path−analysis results indicated that standardized direct path coefficients from both NUpE and NUtE to NUE were significant in all three genotypes. However, the relative magnitudes of the direct path coefficients differed by genotype. NUpE exceeded NUtE in Yunyan 87, NUtE exceeded NUpE in Yueyan 97, and the two were numerically similar in K326 (**Figure 9**). Our path coefficients reflect model-based associations rather than causal effects. They remain informative for partitioning correlations among interrelated traits.

### 4.1 Physiological factors influencing NUpE in flue−cured tobacco

NUpE is defined as the capacity of plants to take up and accumulate nitrogen (Moll et al., 1982). The root system is the principal site for nitrogen uptake. Uptake efficiency is influenced by root biomass, root morphology and root uptake capacity. Larger root biomass and favorable root architecture are associated with greater capacity to acquire soil nitrogen (Cusack et al., 2021). Root vigor reflects the metabolic capacity of roots, including uptake, synthesis, respiration and oxidative activities. It is considered a useful physiological indicator of root uptake capacity (Chen et al., 2020a).

In the present study, NUpE was positively correlated with multiple root performance parameters, including root biomass, root−morphology metrics and root vigor. These correlations were statistically significant (**Figure 9**). Yunyan 87 had lower root biomass, less favorable root morphology and lower root vigor, consistent with its lower NUpE. By contrast, Yueyan 97 and K326 exhibited higher NUpE, with no significant difference between them (*p* > 0.05). However, they employed contrasting root strategies. K326 had larger root biomass and greater total length, surface area and volume. However, it showed relatively lower root vigor. In contrast, Yueyan 97 had higher root vigor with smaller root size. These differences coincided with similar NUpE between the two genotypes.

Xiong et al. (2016) reported comparable patterns in wheat. Zhoumai 28 had greater root biomass and absorbing area, while Zhengmai 366 exhibited higher root vigor. However, nitrogen accumulation did not differ significantly between the two genotypes. This observation is consistent with the notion that lower root vigor may be associated with limited metabolic capacity, which could constrain assimilation and transport of absorbed nitrogen. Taken together, these findings support the view that root biomass, root morphology and root vigor are interrelated factors associated with NUpE.

Flue−cured tobacco preferentially takes up NO_3_^−^ via its root system (Chen et al., 2020b). Only a small fraction is reduced to ammonium (NH_4_^+^) in the roots. Most of the absorbed nitrate is transported to the leaves for assimilation (Hachiya and Sakakibara, 2017). In sugarcane, Hajari et al. (2015) showed that impaired transport of nitrate from roots to shoots limited overall nitrate uptake and reduced NUpE. In contrast, NUtE was less affected in their system. By analogy, measurements of bleeding−sap flow and NO_3_^−^ flux may provide insight into nitrate transport and its potential contribution to NUpE in tobacco.

In the present study, bleeding−sap flow rate and NO_3_^−^ flux were positively correlated with NUpE (**Figure 9**), whereas correlations with NUtE were weak and not significant. Both Yueyan 97 and K326 had significantly higher bleeding−sap flow rates and NO_3_^−^ fluxes than Yunyan 87 (*p* < 0.05; **Figure 3**). Studies in maize have similarly shown that increasing NO_3_^−^ flow rate in bleeding sap can promote nitrogen accumulation in the shoot (Xie et al., 2019). Taken together, these results suggest that bleeding-sap flow and NO_3_^−^ flux are associated factors for NUpE in flue-cured tobacco. But they are correlative from a single site. Causal tests such as ^15^N tracing or manipulations are needed.

NR is a key rate−limiting enzyme in nitrogen assimilation, catalyzing the reduction of NO_3_^−^ to NO_2_^−^.NiR then reduces NO_2_^−^ to NH_4_^+^, which is subsequently assimilated into organic N compounds (Gayatri et al., 2024). NR and NiR activities occur in roots, stems and leaves. However, in many species these activities are higher in leaves, making them the primary sites of NO_3_^−^ assimilation (Andrews and Raven, 2022).

In this study, leaf NR and NiR activities were higher in Yueyan 97 and K326 than in Yunyan 87 during the measured growth stages (**Figure 4A**). Correlation analysis showed a positive association between NUpE and leaf NR/NiR activities (**Figure 9**). These results indicate that genotypes with higher leaf NR and NiR activities tend to accumulate more nitrogen and exhibit higher NUpE. Enhanced reduction of NO_3_^−^ and NO_2_^−^ in leaves may help sustain a favorable concentration gradient. This gradient likely promotes continued root-to-shoot NO_3_^−^ transport and stimulates root uptake (Yu et al., 2021; Kinoshita and Kinoshita, 2022). Wu et al. (2018) similarly emphasized the regulatory role of nitrogen metabolic enzymes in plant N uptake. At the same time, these associations were observed in three genotypes at a single site and without isotopic ^15^N partitioning. Targeted ^15^N assays or genetic/physiological manipulations are needed to establish causality.

Conversely, we observed a weak to moderate negative correlation between leaf NR/NiR activities and NUtE in Yunyan 87 and Yueyan 97 (**Figure 9**). This pattern could occur when increased enzymatic activity is linked to greater plant N accumulation without a proportional rise in dry−matter production. As a result, NUtE decreases (NUtE = yield [dry matter]/total plant N) (Moll et al., 1982). Anas et al. (2020) reported that overexpression of NR or NiR increased N uptake capacity but did not significantly improve biomass or yield in their system, consistent with this interpretation. These inferences are based on correlative data from a single location and a limited number of genotypes. Experimental validation will be required to test the mechanism. Possible approaches include ^15^N tracing, manipulation of enzyme activity, or controlled alterations of C–N balance.

### 4.2 Physiological factors influencing NUtE in flue−cured tobacco

We distinguish “Gross photosynthetic rate” from “carbon-use efficiency” (CUE). Gross photosynthetic rate is the instantaneous carbon assimilation per unit leaf area (*P_gross_*), whereas CUE is the fraction of fixed carbon retained in biomass rather than lost to respiration. At the leaf scale, CUE can be approximated as 1 − *R*/*P_gross_* (Amthor, 2025). In this study, we approximate the instantaneous CUE of flue-cured tobacco leaves as 1 − *R_d, day_*/*P_gross_*.

High NUE and NUtE in crops are often associated with vigorous leaf−level photosynthetic capacity (Guo et al., 2021). However, in the present study Yueyan 97, despite its higher NUE and NUtE, exhibited lower leaf photosynthetic performance than Yunyan 87, which is characterized by lower NUE and NUtE. Specifically, chlorophyll−fluorescence analysis showed that *Fv/Fm* and ΦPSII were lower in Yueyan 97 than in Yunyan 87 (**Figure 6**). These results suggest that Yueyan 97 had lower photochemical efficiency at the leaf level during the measured stages.

Leaf photosynthetic activity is often positively related to leaf N status (Zhong et al., 2024). An increase in whole−plant biomass can dilute tissue N (the “dilution effect”), so that N concentration (mass−based) often decreases as yield or dry matter increases. Consequently, tissue N concentration may be negatively associated with yield or with efficiency metrics that scale with biomass (Isfan, 1993; Zhao et al., 2015; Michel et al., 2019; Arisede et al., 2020). Similarly, studies in sugarcane have shown that genotypes with lower tissue nitrogen concentrations can nonetheless achieve higher whole-plant dry biomass, consistent with greater carbon fixation per unit of plant nitrogen (Leal Junior et al., 2025). In our experiment, Yueyan 97 accumulated more total N than Yunyan 87. However, its leaf N concentration was lower (**Figure 5B**), consistent with a dilution effect. Therefore, the relatively low leaf N concentration in Yueyan 97 may have contributed to its lower net photosynthetic rate.

To further investigate the intriguing observation that Yueyan 97 had a lower net photosynthetic rate yet high NUE and NUtE, we compared *R_d, day_* and *R_d, night_* among three tobacco genotypes. Previous studies estimate that approximately 30% to well over 50% of carbon fixed by photosynthesis is subsequently respired back to the atmosphere (Amthor, 2025). In our experiment, Yueyan 97 exhibited significantly lower rates of both *R_d, day_* and *R_d, night_* than Yunyan 87 (**Figure 7**). Previous work has reported positive associations between leaf respiration rates and tissue N concentration, with respiration often increasing as N concentration rises (Razaq et al., 2017; Thurner et al., 2025).

The lower leaf N concentration observed in Yueyan 97 (**Figure 5B**) may be associated with its lower photosynthesis and respiration rates, although causality cannot be inferred from these correlative data. Lower respiration could decrease the proportion of recently fixed carbon lost to metabolism and is associated with increased soluble sugar content. These sugars can be catabolized to supply additional carbon skeletons for nitrogen assimilation and dry-matter synthesis (Jian et al., 2025), which may help explain the higher NUtE observed in Yueyan 97.

Evidence from other crops supports the idea that lower leaf respiration is linked to higher productivity. Higher-yielding wheat populations have been found to exhibit reduced leaf respiration rates (Pinto et al., 2017). Under high planting density, modern maize hybrids exhibit lower leaf area–specific respiration, reducing carbon loss during critical periods. This reduction allows more florets to survive and set grain (Cagnola et al., 2021). In rice, increased nighttime respiration of the flag leaf is associated with fewer spikelets per panicle and reduced yield (Xu et al., 2021). Together, these studies suggest that lowering respiration can conserve carbon for yield formation and may improve NUtE. The magnitude and consistency of these benefits depend on the species and the environment. To better integrate photosynthesis, respiration, and NUtE, we integrate these results accordingly.

In our study, although Yueyan 97 exhibited a lower leaf net photosynthetic rate (**Figure 5A**) and reduced photochemical performance (**Figure 6**), it consistently maintained a lower *R_d, day_*/*P_gross_* (**Figure 8C**). In addition, it showed lower *R_d, day_* and *R_d, night_* (**Figure 7**), higher leaf C/N, and higher NUtE (**Figure 8A**, **Table 1**). Taken together, these associations point to a carbon-sparing physiological strategy. By decreasing the proportion of assimilated carbon expended on respiration, more carbon is retained per unit plant N. This retained carbon is allocated to growth, thereby supporting higher NUtE even when net photosynthesis is not maximal. In other words, a lower net photosynthetic rate does not necessarily imply lower NUtE. When respiratory costs decline and CUE rises, plants can still achieve a higher carbon-to-nitrogen output ratio. Consistent with this interpretation, we observed a significant positive correlation between C/N and NUtE (**Figure 8B**). These inferences are correlational rather than causal. Nevertheless, they align with established relationships between tissue N and respiration. They also converge with independent evidence in other crops showing that lower leaf-specific respiration is associated with greater biomass or yield (Baruah et al., 2023; Lu et al., 2024). We therefore view the “low respiratory burden–high CUE–high NUtE” phenotype as a plausible and actionable physiological strategy. It is amenable to selection and management optimization, rather than an anomalous observation. These inferences are correlative and single-site. ^15^N/manipulation will be required to test causality and quantify how reduced respiration contributes to NUtE across genotypes and environments.

### 4.3 The contributions of NUpE and NUtE to NUE

We examined the separate contributions of NUpE and NUtE to NUE across three flue−cured tobacco genotypes. We found genotype−dependent differences in these contributions. In Yunyan 87, NUpE contributed more to NUE than NUtE, whereas in Yueyan 97 the opposite pattern was observed. K326 also showed a greater contribution from NUpE than from NUtE, but the difference was smaller than that in Yunyan 87 (**Figure 9**).

These results indicate that, within a species, the relative importance of NUpE and NUtE varies. Similar findings have also been reported in wheat (Xiong et al., 2016). Across crops, the dominant contributor to high NUE appears to vary by species and context. Stahl et al. (2019) reported that NUtE explained more of the variation in NUE in rapeseed, whereas studies in maize (Zhang et al., 2021) and wheat (Huang et al., 2025) found stronger associations between NUE and NUpE. In other words, total plant N accumulation correlated more strongly with yield. Thus, the relative importance of NUpE and NUtE likely reflects crop physiology and breeding history.

Based on our results, improving NUE in flue−cured tobacco will likely benefit from simultaneous enhancement of both NUpE and NUtE. In practice, this could involve breeding varieties that combine efficient N acquisition with efficient conversion of accumulated N into dry matter. It could also involve agronomic practices that target uptake, such as root traits and the timing and placement of N. It could further target utilization, such as carbon fixation per unit N and partitioning. Finally, we note that our quantification of contributions was based on stepwise linear regression using data from a single site and three genotypes. Multi-environment trials and manipulative experiments, including ^15^N tracer validation, would help validate these findings. They would also help determine the most effective breeding or management strategies across diverse environments.

### 4.4 Coordinated framework and practical targets for improving NUE

In summary, our results indicate that NUE can be explained by a coordinated framework of uptake–transport–assimilation–utilization. Higher NUpE co-occurs with larger root biomass and more favorable root morphology, greater root metabolic vigor, and more efficient xylem transport of nitrate. Elevated leaf NR/NiR activity may help sustain a root-to-shoot NO_3_^−^ gradient. Together, these traits promote whole-plant N accumulation. Downstream, at the NUtE level, performance depends less on maximal photosynthetic capacity and more on the cost of respiration. Although the high-NUtE genotype shows relatively lower photosynthesis, it exhibits lower day/night dark respiration and a lower *R_d, day_*/*P_gross_*. This improves carbon use efficiency, retains more carbon per unit plant N, increases leaf C/N, and ultimately raises NUtE. Genotypic differences in NUE arise from two interacting levers: uptake (root acquisition → transport → leaf reduction) and utilization (respiratory cost → CUE). Together, these determine how much N is acquired and how efficiently that N is converted into biomass.

From a breeding and management perspective, nitrogen uptake and utilization should be optimized in concert. For NUpE, prioritize genotypes with greater total root length and surface area and higher root vigor, with particular emphasis on early growth. For rapid field screening, bleeding-sap flow and xylem NO_3_^−^ flux can serve as proxy indicators. For transport–assimilation alignment, maintain leaf NR/NiR at moderate-to-high levels that match transport capacity. Avoid excessive reduction, which yields no biomass gain. For NUtE, aim to lower day/night dark respiration per unit leaf area and reduce *R_d, day_*/*P_gross_* to enhance carbon use efficiency (CUE). Monitor these traits with streamlined gas-exchange and chlorophyll fluorescence spot checks. In practice, an effective bundle comprises efficient roots, robust NO_3_^−^ transport, appropriately calibrated NR/NiR activity, and low leaf-specific respiration. At the same time, align N application rates and timing with root activity rhythms to avoid late-season over-fertilization that undermines efficiency. Our results were obtained at a single location and season with three genotypes and are primarily correlative. Future work should extend to multi-site, multi-year trials with more genotypes. They should also include ^15^N tracing and targeted perturbations to test causality and generalize recommendations.

## 5 Conclusion

This study systematically compared the nitrogen efficiency of three flue−cured tobacco genotypes (Yunyan 87, Yueyan 97 and K326) across different N application rates. We found that Yunyan 87 had relatively lower NUpE, NUtE and overall NUE than the other genotypes, whereas Yueyan 97 exhibited the highest NUpE, NUtE and NUE. K326 displayed high NUpE but low NUtE, resulting in intermediate NUE (**Table 1**). Comparative analysis indicated that factors associated with NUpE included root system biomass and architecture, root vigor, xylem sap flow rate and nitrate flux in sap, and leaf nitrogen metabolism enzyme activities. NUtE was related to both net photosynthetic rate and respiration rate. Higher NUtE was more strongly associated with reduced respiratory carbon loss than with increased photosynthetic rate. Both NUpE and NUtE contributed significantly to NUE within each genotype. The relative contributions of NUpE versus NUtE to NUE differed by genotype. NUpE contributed more than NUtE in Yunyan 87, NUtE contributed more in Yueyan 97, and the two contributions were numerically similar in K326 (**Figure 9**). These findings suggest that improving NUE in flue−cured tobacco may benefit from concurrent enhancement of N acquisition and N utilization traits through targeted breeding and management practices. While path coefficients are not causal effects, they help clarify the relative strength and direction of associations.

Limitations include the single location/season, the limited number of genotypes, and the correlative nature of the data without ^15^N or manipulation-based validation. Future studies should extend to multi-site, multi-year, ^15^N-enabled trials and use mechanistic perturbations to test causality and refine recommendations.

## Data Availability

The original contributions presented in the study are included in the article/supplementary material. Further inquiries can be directed to the corresponding authors.
